# Identification of Potential Biomarkers in Prostate Cancer Microarray Gene Expression Leveraging Explainable Machine Learning Classifiers

**DOI:** 10.3390/cancers17233853

**Published:** 2025-11-30

**Authors:** Ahmed Al Marouf, Jon George Rokne, Reda Alhajj

**Affiliations:** 1 Department of Computer Science, University of Calgary, Calgary, AB T2N 1N4, Canada; rokne@ucalgary.ca; 2 Department of Computer Engineering, Istanbul Medipol University, Istanbul 34810, Turkey; 3 Department of Health Informatics, University of Southern Denmark, 5230 Odense, Denmark

**Keywords:** explainable machine learning, prostate cancer, microarray data, biomarker identification, random forest

## Abstract

**Background and Objective:** Prostate cancer remains one of the most prevalent and potentially lethal malignancies among men worldwide, and timely and accurate diagnosis, along with the stratification of patients by disease severity, is critical for personalized treatment and improved outcomes for this cancer. One of the tools used for diagnosis is bioinformatics. However, traditional biomarker discovery methods often lack transparency and interpretability, which means that clinicians find it difficult to trust biomarkers for their application in a clinical setting. **Methods:** This paper introduces a novel approach that leverages Explainable Machine Learning (XML) techniques to identify and prioritize biomarkers associated with different levels of severity of prostate cancer. The proposed XML approach presented in this study incorporates some traditional machine learning (ML) algorithms with transparent models to facilitate understanding of the importance of the characteristics for bioinformatics analysis, allowing for more informed clinical decisions. The proposed method contains the implementation of several ML classifiers, such as Naive Bayes (NB), Random Forest (RF), Decision Tree (DT), Support Vector Machine (SVM), Logistic Regression (LR), and Bagging (Bg); followed by SHAPly values for the XML pipeline. In this study, for pre-processing of missing values, imputation was applied; SMOTE (Synthetic Minority Oversampling Technique) and the Tomek link method were applied to handle the class imbalance problem. The k-fold stratified validation of machine learning (ML) models and SHAP values (SHapley Additive explanations) were used for explainability. **Results:** This study utilized a novel tissue microarray data set that has 102 patient data comprising prostate cancer and healthy patients. The proposed model satisfactorily identifies genes as biomarkers, with highest accuracy obtained being 81.01% using RF. The top 10 potential biomarkers identified in this study are DEGS1, HPN, ERG, CFD, TMPRSS2, PDLIM5, XBP1, AJAP1, NPM1 and C7. **Conclusions:** As XML continues to unravel the complexities within prostate cancer datasets, the identification of severity-specific biomarkers is poised at the forefront of precision oncology. This integration paves the way for targeted interventions, improving patient outcomes, and heralding a new era of individualized care in the fight against prostate cancer.

## Simple Summary

Prostate cancer is one of the most common and deadly cancers in men. Accurate diagnosis and determining disease severity are essential for personalized treatment and better outcomes. This study introduces an Explainable Machine Learning (XML) approach to identify biomarkers linked to different severity levels of prostate cancer, addressing the lack of transparency in traditional bioinformatics methods. The proposed framework combines conventional machine learning algorithms with SHAP values for model explainability. Using a novel tissue microarray dataset of 102 prostate cancer and healthy patients, the Random Forest model achieved the highest accuracy of 81.01%. The top 10 potential biomarkers identified in this study are DEGS1, HPN, ERG, CFD, TMPRSS2, PDLIM5, XBP1, AJAP1, NPM1 and C7. The results demonstrate that XML can enhance biomarker discovery and interpretability, supporting more transparent and personalized clinical decision-making. This approach marks a promising step toward precision oncology and individualized prostate cancer care.

## 1. Introduction

Cancer has been proven to be a sophisticated and heterogeneous disease that faces significant challenges in its understanding, diagnosis, and treatment. Progress in regards to these challenges depends on collecting data from which information can be extracted. In this regard, recent advances in data collection and storage have enabled the development of large collections of biomedical cancer data. However, processing these data also presents challenges. Fortunately, the concurrent emergence of sophisticated computational tools based on Machine Learning (ML) has led to promising approaches to extract meaningful insights from vast and intricate biomedical data sources.

The inherent opacity of many ML algorithms raises concerns about their reliability and interpretability within critical domains such as healthcare and specifically for cancers. To address the possible solution to the challenges mentioned above, explainable machine learning (XML) has appeared to be a crucial path forward for healthcare and cancer research, with the aim of unraveling the intricate decision-making processes of ML models, thus improving their interpretability and trustworthiness [1]. ML-based models, particularly deep learning algorithms, have achieved remarkable accuracy for tasks like tumor classification, survival prediction, and treatment response assessment, leveraging diverse datasets encompassing genomics, imaging, and clinical records. However, as noted above, their black-box nature hampers their widespread adoption by clinicians for which comprehensible and transparent decision making is of paramount importance [2].

XML tools, such as the SHAP value-based method, SHAP (SHapley Additive Explanations) [1], Agnostic models, namely, LIME (Local Interpretable Model-agnostic explanations) [3], and others, offer avenues for understanding and interpreting the predictions made by complex ML models. For example, LIME provides local interpretations by approximating the behavior of an ML model around specific instances, thus facilitating the identification of features crucial to individual predictions. Similarly, SHAP values [1] quantify the importance of features used in model predictions, and thus provide information on their relative importance. Moreover, studies have highlighted the utility of XML in elucidating the underlying biological mechanisms that drive cancer progression and its response to therapies [4]. For example, researchers have identified key genomic alterations associated with drug resistance in specific cancer types employing XML techniques, unraveling intricate gene interactions that govern treatment responses. These advances in XML have generated considerable interest in both academia and clinical practice, as academic researchers and healthcare professionals recognize the significance of transparent and interpretable ML models for enhanced clinical decision making, understanding disease mechanisms, and identifying novel therapeutic targets.

The detection and stratification of severity levels for prostate cancer is imperative due to the disease’s heterogeneity and varied clinical courses, necessitating tailored treatment strategies. The reason is that an accurate assessment of severity guides clinicians when making decisions regarding intervention or not. This can then minimize overtreatment for less severe forms while ensuring appropriate management for aggressive forms. Hence, together with early detection, accurate differentiation between low-risk and high-risk prostate cancer cases means that unnecessary invasive procedures for indolent tumors can be avoided while prioritizing aggressive therapies for potentially life-threatening malignancies.

The research by Cooperberg et al. [5] underscores the importance of risk stratification in the management of prostate cancer, emphasizing the role of risk assessment tools in guiding treatment decisions and improving patient outcomes. In addition, they emphasize that the identification of high-risk cases allows timely interventions, potentially curbing disease progression, and improving long-term survival rates. This is also noted in the work of Loeb et al. [6], which highlights the importance of risk stratification in reducing prostate cancer mortality by targeting aggressive tumors.

Kraujalis et al. [7] propose machine learning-based mortality estimation models for prostate cancer patients and demonstrate that predictive modeling can assist clinicians by identifying individuals at higher risk of adverse outcomes. Their study shows that ML models can improve accuracy compared to traditional statistical approaches, reinforcing the prognostic value of data-driven methods in oncology. In contrast, Subrahmanya et al. [8] provide a broader perspective on how data science transforms healthcare systems—highlighting applications such as clinical decision support, predictive analytics, and personalized treatment planning. Together, these studies emphasize that machine learning not only enhances outcome prediction [7] but also contributes to large-scale healthcare improvements, supporting more reliable and personalized medical decision-making [8].

Severity detection also helps to evaluate the need and timing of interventions. It also helps to understand the spectrum from actively monitoring low-risk cases to high-risk cases, which may lead to specific treatments, such as surgery or the use of chemotherapy, for high-risk scenarios. Proper risk stratification is also crucial to avoid unnecessary treatments and their associated adverse effects, which is also supported by the work of Dahabreh et al. [9], who also advocate for risk-based treatment decisions for prostate cancer to optimize clinical outcomes while minimizing unnecessary procedures, improving patient quality of life.

Hence, accurate detection and stratification of prostate cancer severity levels are fundamental when tailoring treatment approaches, optimizing patient outcomes, and mitigating risks associated with both overtreatment and undertreatment. This stratification aligns with the paradigm of precision medicine, ensuring that interventions are tailored to individual patients based on the specific characteristics and aggressiveness of their disease.

In this study, the primary objective is to identify prostate cancer biomarkers or gene signatures from high-dimensional microarray data. Multiple traditional machine learning (ML) algorithms are applied to determine the most discriminative genes associated with different cancer risk levels. These ML models are chosen due to their proven effectiveness in classification and prediction tasks involving multiclass data. To enhance model transparency, the ML workflow is further extended into an explainable machine learning (XML) pipeline, allowing improved interpretability and clarity in feature importance.

The following section presents an overview of the clinical severity levels used to categorize prostate cancer patients. Details regarding the publicly available dataset and the associated pre-processing steps are also provided. A comprehensive results section reports all experimental outcomes at each stage of the analysis. Finally, the paper concludes by summarizing the key findings and emphasizing how the approach contributes to prostate cancer biomarker discovery and clinical interpretability.

## 2. Materials and Methods

The methods section provides a description of the proposed XML pipeline, as well as the data sets used and the performance metrics applied. The usability and limitations of the proposed approach are also considered.

### 2.1. Proposed Methodology

**Figure 1:**
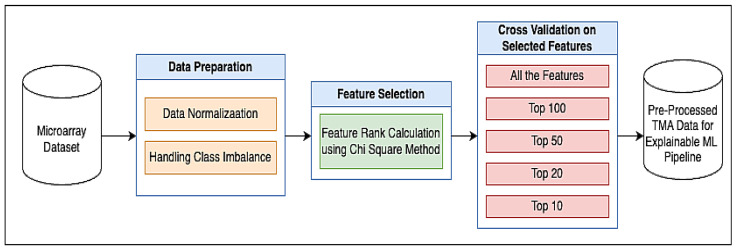
Proposed methodology for biomarker identification.

An XML pipeline has been constructed, as shown in Figure 1. This pipeline integrates the performance of various ML models through sequential stages, starting with data pre-processing. The processed data are sent to the ML model training, model validation, testing, and quantification.

**Figure 2:**
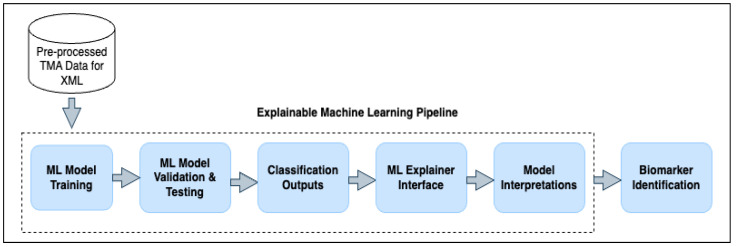
Proposed explainable machine learning pipeline for prostate cancer biomarker identification.

Each stage is detailed in the following subsections, with the overall pipeline architecture shown in Figure 2. The study compares multiple ML algorithms, including NB, SVM, DT and LR, alongside tree ensemble-based methods such as RF and BG, to evaluate their effectiveness within the XML framework. This comprehensive approach enables systematic assessment and comparison of model performance.

Naive Bayes (NB) is computationally efficient and scales well with increasing feature dimensionality, making it particularly effective for high-dimensional datasets. Its simplicity in implementation and interpretability make it an excellent choice for rapid prototyping and establishing baseline performance. Additionally, NB naturally supports multiclass classification without requiring any modifications or additional techniques.

Decision trees (DT) offer high interpretability, allowing users to easily understand and explain the rationale behind classification decisions [10]. They provide valuable insights into feature importance, which aids in feature selection and deepens understanding of the underlying data. Furthermore, decision trees can model complex, nonlinear relationships between features and the target variable.

Support Vector Machines (SVMs) excel in data with higher-dimensional spaces, making them a good fit for problems with many features [11]. To tackle the linear and non-linear types of relationships more effectively, multiple kernel functions could be employed in SVM. Their objective of maximizing the margin between classes often results in improved generalization and robust performance [11].

Logistic regression (LR) offers probabilistic interpretations of classification outcomes, providing nuanced insights into prediction confidence [12]. It can be regularized to reduce overfitting and improve resilience to noise and outliers. Logistic regression is also efficient in terms of computation and can manage large datasets while using relatively little memory.

The Random Forest (RF) is an ensemble method that aggregates the results of several decision trees to enhance predictive precision. By aggregating the output of many trees through voting or averaging, RF reduces variance and the risk of overfitting, resulting in more robust and accurate classifications [13]. It is highly effective in handling high-dimensional feature spaces and large datasets. The random feature selection of the method for each tree promotes diversity between trees, further improving generalization and mitigating overfitting.

It is also robust and can handle noisy data and outliers. Since it constructs multiple trees based on random subsets of the data and features, the impact of individual noisy data points is reduced, leading to a more robust classification. RF can also provide valuable information on the importance of features. By analyzing the frequency with which features are selected for splitting across multiple trees, it can identify the most informative features for classification, aiding in feature selection and understanding the underlying data patterns.

It can be easily parallelized, allowing efficient training on large datasets. Since each tree in the ensemble can be trained independently, Random Forest implementations can take advantage of parallel processing to speed up the training process.

Python classifier packages, such as those in scikit-learn, have been used to implement each of the machine learning methods. During model development, stratified k-fold cross-validation was used to ensure that each fold maintains the same class distribution as the full data set, improving the reliability of performance estimates, especially in unbalanced classification problems. The pipeline architecture enables easy swapping and evaluation of different models, facilitating direct comparison of their performance metrics. Detailed descriptions of each component of the methodology are provided in the following subsections.

#### 2.1.1. Dataset

The data set for the study contained prostate cancer microarray data, which is publicly available on the MIT website. The dataset was first introduced by Singh et al. [14]. The data set consists of two groups, prostate tumor samples and normal prostate samples. The training set has nearly equal numbers of both types, totaling around 102 samples with approximately 12,600 genes. To identify significant gene expression differences associated with the clinical and pathological characteristics of prostate cancer, researchers analyzed 235 prostate tissue samples collected from patients who underwent surgery between 1995 and 1997 [14]. There is a separate testing set from a different study, which has significantly lower overall gene expression levels compared to the training set. The high dimensionality of the data is one of the main challenges in using this data set. Along with the “curse of dimensionality”, the class imbalance problem is also present in the dataset. Therefore, the raw data has to go through the data preparation step (explained in next section) before loading them into explainable machine learning pipelines. The details of the raw data set are presented in Table 1. More statistical details on clinical and pathological features can be found in the original article [14]. The data set can be found in the Gene Expression Omnibus (GEO) databank (https://www.ncbi.nlm.nih.gov/geo/query/acc.cgi, accessed on 12 November 2025) with GEO accession number GSE68907.

#### 2.1.2. Data Preparation

Data for this research were obtained from laboratory immunohistochemical tests and systematically recorded in spreadsheets, organized by each patient’s identification. Multiple samples may be taken from different parts of the tumor or different tumors for a given patient, resulting in multiple rows with the same ID with diverse test results. This means that the spreadsheets can not be used for direct input into machine learning pipelines. Therefore, pre-processing techniques that involve normalizing gene expression data have been applied to ensure compatibility with the machine learning pipeline without altering the original information.

##### Data Normalization

The data have variations, making it difficult to interpret by a machine learning model. To address this, the data are standardized so that all patients have the same format. This involves simplifying the inputs into categories based on gene signatures.

##### Handling Class Imbalance Problem Using the SMOTE-Tomek Link Method

In addition to missing values, the merged dataset suffers from class imbalance. To address this, the Synthetic Minority Oversampling Technique (SMOTE) [15] was combined alongside the Tomek link method. SMOTE generates synthetic minority class samples by interpolating between nearest minority neighbors, rather than duplicating existing examples, thus enhancing minority class representation. Tomek links [16], a modification of condensed nearest neighbors, identify pairs of samples from opposite classes that are each other’s nearest neighbors; majority class samples in these pairs are removed to clean class boundaries. Combining SMOTE (oversampling) with Tomek links (undersampling) leverages the strengths of both methods, producing a cleaner, more balanced dataset that improves model learning and class separability.

#### 2.1.3. Applying Machine Learning Methods

The machine learning methods used in this study included NB [17], DT [10], SVM [11] and LR [12]. In addition, ensemble techniques such as RF [13] and BG [18] were used to improve the performance of the model by combining multiple learners. The sklearn python classifier packages were taken to develop these machine learning methods. Stratified k-fold cross-validation was also applied with these methods. A pipeline architecture was used, which allowed easy integration and experimentation with different models to assess the performance measures for each one. Furthermore, the results of the ML methods were compared to find the best performing method, and the following explainable machine learning concepts were developed based on the best performing ML method.

#### 2.1.4. Chi-Square Method for Feature Selection

The Chi-square method is a widely used statistical test method, which is used for feature selection purposes, particularly in a classification problem with categorical feature and target variables. The measurement used for the method calculates the dependence between a feature of the model and the target class. Hence, the most relevant features for the model could be identified. The chi-square statistic is calculated using Equation (1).

(1) $$\chi^{2}=\sum \frac{{\left(O-E\right)}^{2}}{E}$$

In Equation (1), *O* represents the observed frequency in the contingency table and *E* represents the expected frequency assuming independence. Interpreting the score $\chi^{2}$ is easy to understand the relationship between features and the target class. The higher $\chi^{2}$ score suggests a strong relationship between the feature and the class, whereas the lower score suggests that there is little relationship between them.

#### 2.1.5. Explainable Machine Learning Pipeline

The proposed explainable machine learning (XML) pipeline consists of the training, validation, and testing of the ML model and an explanation interface. For most classification problems, the training–testing–validation sequence is the common steps to be followed. In the proposed model, one step ahead is used for the classification output findings as a post-processing component. The output results are fetched into the ML-explainer interface, which takes account of the best or most effective features to explain the cases. The results are used in both local and global interpretations.

Explainability encompasses two main levels, namely local explainability and global explainability, and for more details, see [19]. Local explainability focuses on explaining the individual predictions of a machine learning learning algorithm, whereas global explainability elucidates the ultimate decision across all data points, offering a comprehensive analysis in terms of overall fidelity. It exclusively delineates the instance level, underscoring the significance of this aspect [20]. For explaining these components, the SHAP (SHapley Additive Explanations) proposed by Lundberg and Lee [1] was used. SHAP is a model-agnostic interpretation technique designed to provide explanations for predictions for any machine learning model, regardless of the underlying algorithm. Hence, these methods are versatile and can be applied to various types of model, such as decision trees, random forests, support vector machines, neural networks, etc. A brief introduction to SHAP is provided below to form a background for the research.

SHAP aims to clarify the prediction for a given instance, x, by determining the impact of each feature on that prediction. Shapley values are calculated using principles from coalition-based game theory (see [21]), where the feature values of a data instance function as players forming a coalition. Shapley values provide guidance on how to equitably allocate the “payout” (i.e., the prediction) among the features. SHAP specifies the explanation using the formula:

(2) $$g\left(z^{\prime }\right)=\phi_{0}+\sum_{j=1}^{M}\phi_{j}z_{j}^{\prime }$$

Here, the explanation model is denoted by *g*, the coalition vector $z^{\prime }$ has the spaces as $z^{\prime }\;\epsilon \;{\left\{\begin{array}{c} 0,1 \end{array}\right\}}^{M}$, and the maximum coalition size is denoted by *M*. $\phi_{j}\;\epsilon \;R$ is the feature attribution for a feature *j*, the Shapley values.

It turns out that the Random Forest (RF) classifier, which is a tree-based method, was the best performing method among the applied machine learning methods. The RF is an ensemble method that combines multiple models, in this case, multiple decision trees, to improve robustness and accuracy over a single model. The adaptability for overfitting compared to individual decision trees is another reason to obtain higher prediction capabilities. To handle the robustness to noise and outliers in the data, multiple trees are generated in the RF model to address the complexity of the dataset. Compared to the other applied ML methods, RF has been chosen for the explainable pipeline because it demonstrated the highest accuracy.

TreeSHAP proposed by Lundberg et al. [22] in 2018 was used to improve explainability. It was developed for tree-based ML models such as DT, RF, and XGBoost, which works faster, and it is a model-specific alternative. TreeSHAP uses the conditional expectation $E_{X_{j}|X_{-j}}\left(\hat{f}\left(x\right)|x_{j}\right)$ instead of the marginal expectation. This helps to better explain the results of the random forest.

SHAP feature importance analysis [1] was performed to understand the importance of each feature used in the machine learning model. By attributing an importance value to each feature in every prediction, SHAP values offer a localized and consistent explanation of the behavior of the model. These values reveal the characteristics that exert the most influence on a particular prediction, be they positive or negative. In the results section, they have been illustrated using the SHAP feature importance plot generated by SHAP for global interpretation. In addition, beeswarm summary plots [1] have been used to explain the global representation of the features and for local interpretation instances or patient-based force plots. The context for using these relative graphs was explained by Christoph Molnar in [23].

### 2.2. Quantifying Performances

The performance of the machine learning methods was evaluated using precision, recall, *F*1-*score*, and accuracy. The ML pipeline allowed us to optimize for the highest accuracy while also considering other key metrics such as precision, recall, and *F*1 *score*. These parameters were calculated using the following formulas:

(3) $$Precision=\frac{TP}{TP+FP}$$

(4) $$Recall=\frac{TP}{TP+FN}$$

(5) $$F1-score=\frac{2(Precision\times Recall)}{\left(Precision+Recall\right)}$$

(6) $$Accuracy=\frac{TP+TN}{TP+TN+FP+FN}$$

(7) $$SD=\sqrt{\frac{\sum {\left|x-\bar{x}\right|}^{2}}{n}}$$

(8) $$MCC=\frac{TP\times TN-FP\times FN}{\sqrt{\left(TP+FP)\times (TP+FN)\times (TN+FP)\times (TN+FN\right)}}$$

Here, true positive (TP) denotes the fraction of results in which the risk level is actually at the same level as predicted. True negative (TN) denotes the fraction of results in which the risk level is not actually the predicted level. Similarly, false positive (FP) means that the fraction of results where the risk level does not actaully exist, and false negative (FN) is the fraction of results that does not predict the risk level when it is actually at that level. The Matthews correlation coefficient (MCC) is a statistical tool that measures the difference between predicted and actual values. It is useful for evaluating the performance of a binary classifier, especially when the class distribution is imbalanced or when true negatives are important. The *MCC* can range from −1 to +1, with the following interpretations: +1 means perfect prediction, 0 means that it is not better than random prediction, and −1 means total disagreement between predicted and actual value. All the codes related to the explainable machine learning pipeline could be found in this GitHub repository, version v1.0.0, Commit number fd87482, (https://github.com/AhmedAlMarouf/XMLonPCa, accessed on 23 November 2025).

## 3. Results

This section provides the experimental findings accompanied by an in-depth discussion and analysis. The result section has been divided into two main parts: comparing the performances of applied machine learning methods and the interpretations of the results using an explainable machine learning pipeline. For the overall implementation of the methods, Python, version 3.14.0 packages were utilized. In addition to pandas, version 2.3.3 [24], NumPy, version 2.3.5 [25], Matplotlib, version 3.10.7 [26], and re (regular expression), built-in version [27] packages for manipulating the data frames, the Python scikit-learn, version 1.7.2 package [28] has been used for implementing machine learning methods. The regular hyper-parameters have been used for the ML methods. All the codes related to the experiments could be found in this GitHub repository, version v1.0.0, Commit number fd87482 (https://github.com/AhmedAlMarouf/XMLonPCa), accessed on 12 November 2025.

### 3.1. Performance Comparison of Applied Machine Learning Methods

The SMOTE-Tomek link method applied in this study has been used successfully in several previous works [29,30]. For example, Zeng et al. [29] used this integrated approach to handle imbalanced medical data sets to forecast disease progression. Batista et al. [30] combined these two techniques to find the balance between the raw data for the annotation of keywords. Drawing on this established background facilitated the effective application of the combined SMOTE and Tomek link methods for data balancing within our machine learning pipeline.

**Figure 3:**
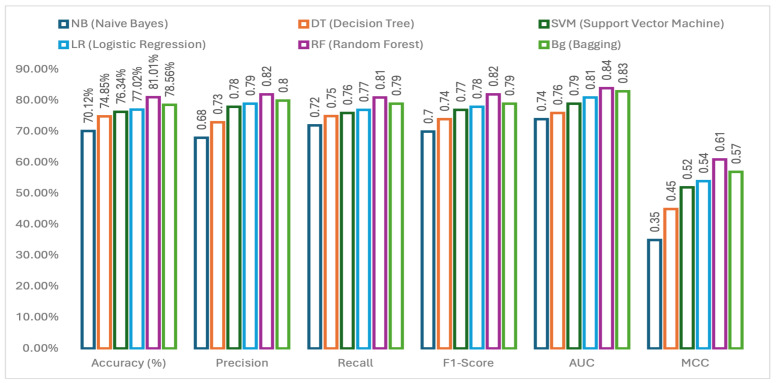
Performance comparison of applied ML methods. Based on six performance metrics (accuracy, precision, recall, f1-score, AUC and MCC (from left to right)) the side-by-side bars represent the performance of each ML model. In all the cases, RF outperformed others by obtaining highest values.

From Figure 3, we can see that the Random Forest (RF) has outperformed the other applied machine learning models. The choice of machine learning models was made based on their applicability to multiclass classification in other research studies. Therefore, among the six applied methods, RF got the highest accuracy of 81.01% and NB got the lowest accuracy of 70.12%. In comparison of ML methods based on other performance metrics, it is evident that RF has done well with these gene data and therefore for the further analysis (i.e., applying imputation techniques, applying feature selection method and explainable methods) RF is chosen. All the methods shown in Figure 3 have been applied using all gene expressions available in the data set. None of the genes were removed for these predictions and a 10-fold cross-validation was applied.

Table 2 presents the performance metrics of the ML pipeline using four different imputation techniques. To evaluate the variability among these methods, imputation was performed using mean, mode (most frequent), median, and constant value strategies in combination with the RF method. Precision, recall, F1 score, and accuracy are presented along with their respective standard deviation values. The most frequent (mode) imputation method achieved the highest obtained accuracy (81.01%), highlighted in bold. Standard deviations are provided in parentheses for each metric to indicate variability.

### 3.2. Experiments on Feature Selection

As mentioned in the previous section, the chi-square test has been performed on the features or genes to handle the high dimensionality of the data. We have setup five different experiments (F-ALL, F-10, F-20, F-50, and F-100) based on the number of genes to be used by RF (in this case). The top genes have been extracted based on the selection of the chi-square method for k best features.

- F-ALL: Experiment with all genes.
- F-10: Experiment with the top 10 genes.
- F-20: Experiment with the top 20 genes.
- F-50: Experiment with the 50 best genes.
- F-100: Experiment with the top 100 genes.

**Figure 4:**
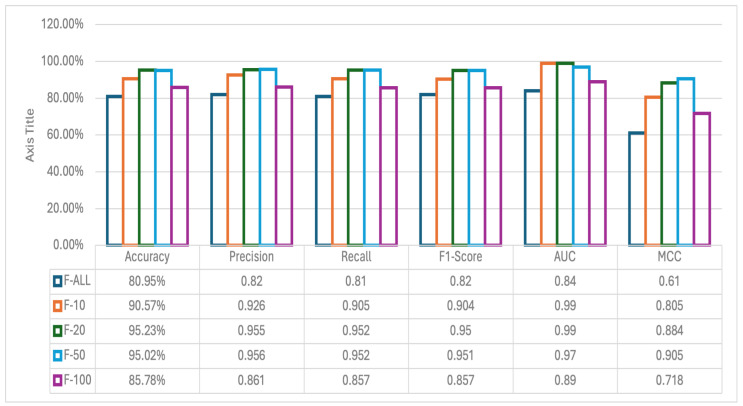
Performance metrics of RF model for feature selection experiments. In five different feature setups (F-ALL, F-10, F-20, F-50 and F-100), the highest accuracy obtained is 95.23% with 20 features. Increasing number of features decreases the overall performance.

Based on the previous experiment of using different machine learning classifiers, we have decided to go further with the implementation of the RF method. Therefore, for each of the above-mentioned experiments, we have chosen RF as the ML method, and the performance metrics have been calculated. The metrics are shown in Figure 4.

**Figure 5:**
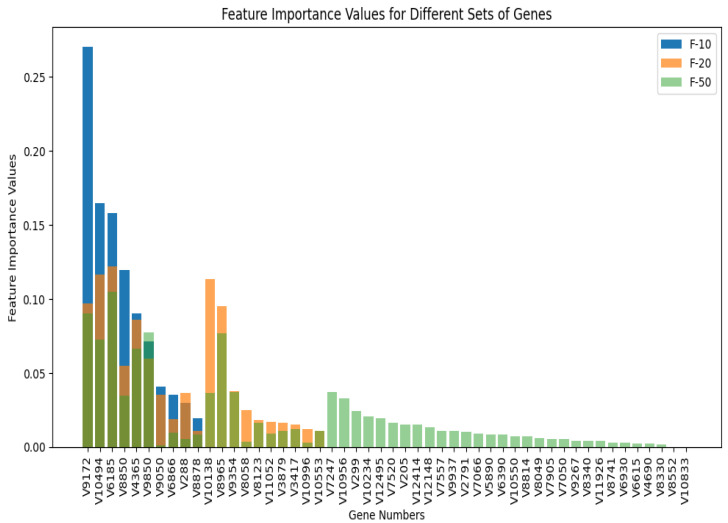
Feature important values for F-10, F-20 and F-50 set of genes. Three different colors are used: blue for F-10, orange for F-20 and light green for F-50. Starting from the V9172 gene, the first 10 genes have been selected for all three experiments. While genes overlapped in all three experiments, orange, blue and light green shows a dark green color. When F-10 and F-20 overlapped, the dark orange color resulted by combining the blue and orange color. Few genes overlapped in F-20 and F-50 from V10138 till V10996. The vector numbers are used in the figure. The gene mapping could be found in the original data files in [14].

**Figure 6:**
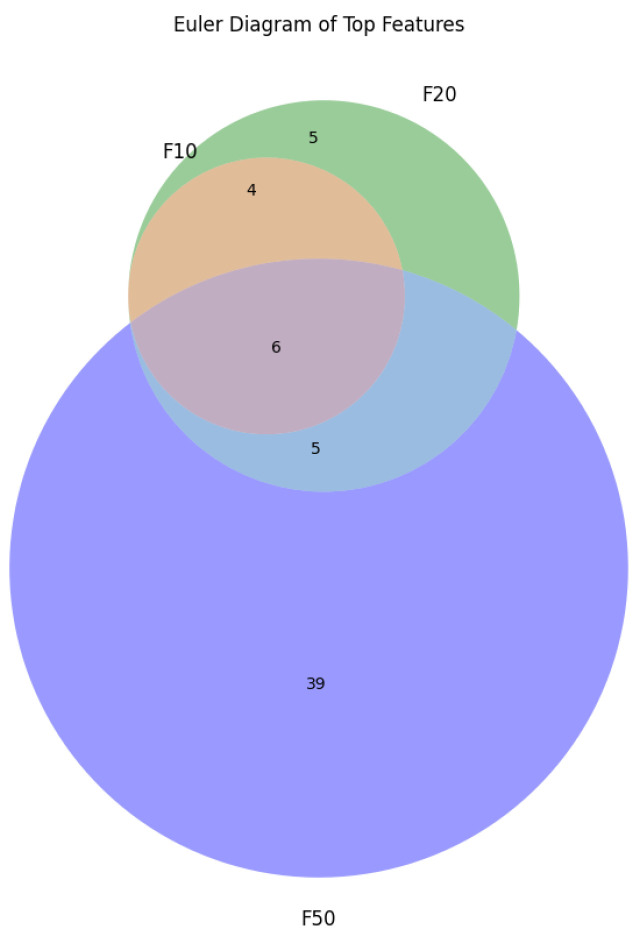
Euler diagram for F-10, F-20 and F-50 set of genes. Orange circle represents F-10, green circle represents F-20 and violet circle represents F-50. The numbers inside the circles define the number of common and different genes among the sets. Overlapping between the feature sets are also shown.

From Figure 4 we can see that using all the genes, aka features, the accuracy of the model is the lowest we get. The highest accuracy is obtained using the top 20 features. The overall performance of the other metrics (precision, recall, F1 score, AUC, and MCC) in F-20 also outperforms the other experiments. The list of features selected for each experiment along with the importance of the feature is shown in Figure 5. We can clearly see some overlap on the selected genes based on the chi-square method. To illustrate overlapping, the Euler diagram in Figure 6 has been used.

### 3.3. Outcome Interpretations Using Explainable Machine Learning Pipeline

According to the proposed methodology, the post-processing technique has been adopted and the best performing ML method (Random Forest) has been kept as the core of the XML pipeline. The proposed XML pipeline only included the random forest method, and the outcome was interpreted using the SHAP, version 0.46.0 XML tool (https://pypi.org/project/shap/, accessed on 23 November 2025) [1,22].

#### 3.3.1. Global Interpretation—Feature Importance

For explaining the global interpretation of the XML pipeline, the feature importance plot for the two classes (class labels: tumor and normal) as well as a beeswarm summary plot. The concept of SHAP feature importance plot is fairly simple. SHAP feature importance plots typically display features ranked by their importance. The importance is measured by the mean absolute SHAP values (using the Formula (9)) in all instances of the data set. Features higher up in the plot are more important in influencing the model’s predictions.

(9) $$I_{j}=\frac{1}{n}\sum_{i=1}^{n}\left|\Phi_{j}^{\left(i\right)}\right|$$

**Figure 7:**
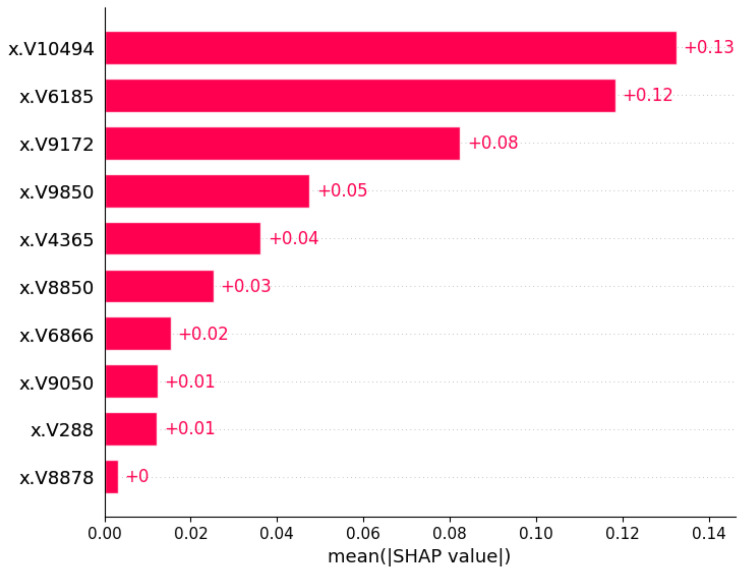
SHAP feature importance plot for top 10 genes. The V-ID used in the vector representation for the XML model. The top genes are DEGS1, HPN, ERG, CFD, TMPRSS2, PDLIM5, XBP1, AJAP1, NPM1 and C7, sequentially from top to bottom.

Figure 7 shows the importance of the SHAP feature for the selected ML method (Random Forest) based on the performance found. Using the Chi-square method for feature/gene selection, the top 10 genes were taken to find the average Shap value and compare them. The V10494 gene received the highest positive 0.13 shap value, whereas the V8878 gene did not receive a shap value at all.

**Figure 8:**
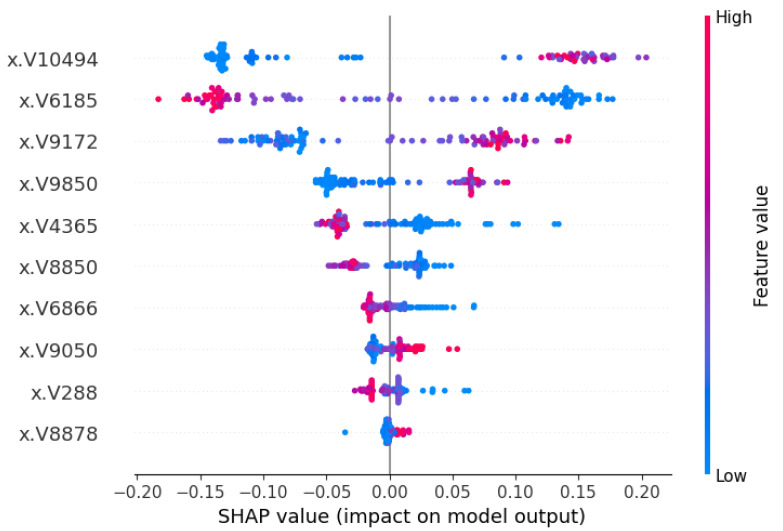
SHAP beeswarm summary plot with feature values for the patients (tumor class). The V-ID used in the vector representation for the XML model. The top genes are DEGS1, HPN, ERG, CFD, TMPRSS2, PDLIM5, XBP1, AJAP1, NPM1 and C7, sequentially from top to bottom.

**Figure 9:**
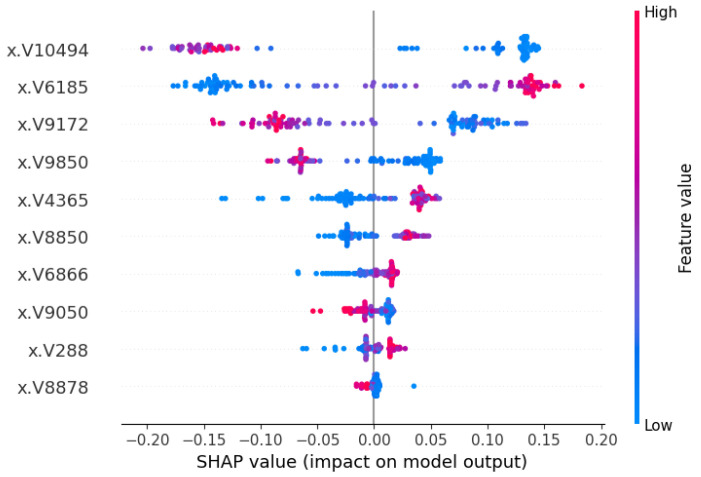
SHAP beeswarm summary plot with feature values for the patients (normal class). The V-ID used in the vector representation for the XML model. The top genes are DEGS1, HPN, ERG, CFD, TMPRSS2, PDLIM5, XBP1, AJAP1, NPM1 and C7, sequentially from top to bottom.

The SHAP beeswarm summary plot introduced in [1] is also considered a vital plot to explain global interpretations. The summary plot combines feature importance with feature impact, where each point represents a Shapley value computed for a specific feature and data instance. In this case, each of the clinical and signature characteristics and parameters/subjects were used to calculate the values. In Figure 8 and Figure 9, the summary plot of the SHAP beeswarm has been demonstrated, mentioning the characteristic values. In Figure 8, the position along the y-axis corresponds to the feature, while the Shapley value on the x-axis determines the placement of the signature value. The color red represents the high value and blue represents the low value. Among the genes, V10494, V6185 and V9172 have more diverse SHAP values, hence they have more impact on the model output. Figure 9 shows similar findings in terms of SHAP values and the impact on the machine learning model for the normal class.

**Figure 10:**
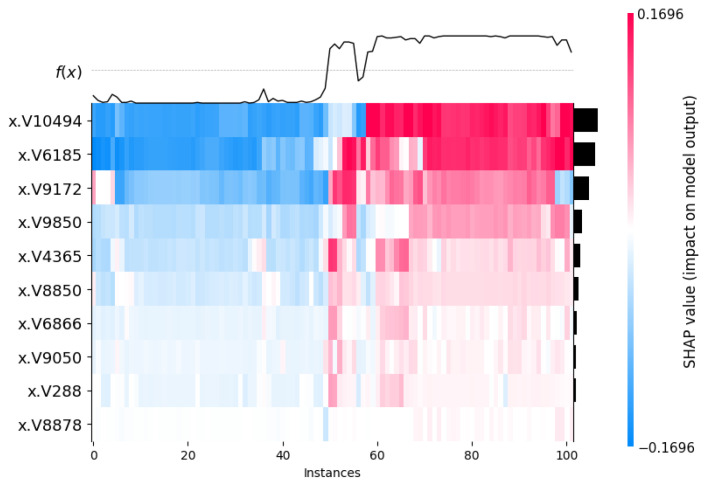
SHAP heatmap plot with top 10 genes on 102 instances (tumor class). The V-ID used in the vector representation for the XML model. The top genes are DEGS1, HPN, ERG, CFD, TMPRSS2, PDLIM5, XBP1, AJAP1, NPM1 and C7, sequentially from top to bottom. The black bars are the visual markers showing the most important feature/genes and used to sort them from higher to lower values.

**Figure 11:**
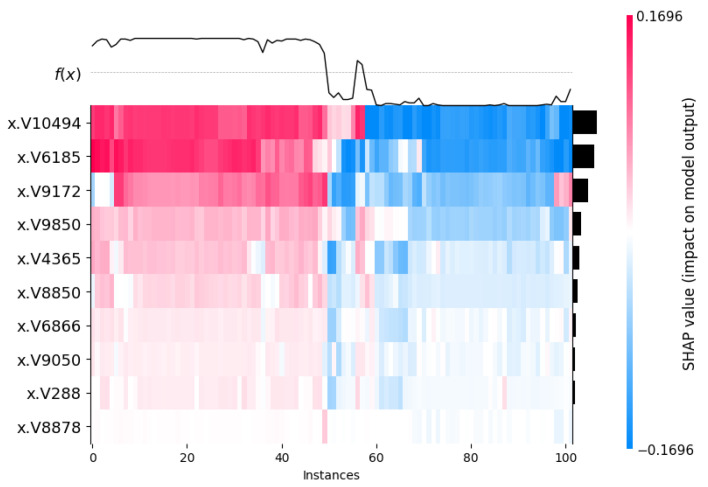
SHAP Heatmap plot with top 10 genes on 102 instances (normal class). The V-ID used in the vector representation for the XML model. The top genes are DEGS1, HPN, ERG, CFD, TMPRSS2, PDLIM5, XBP1, AJAP1, NPM1 and C7, sequentially from top to bottom. The black bars are the visual markers showing the most important feature/genes and used to sort them from higher to lower values.

The heat maps illustrated in Figure 10 and Figure 11 show the impact of the model using the SHAP values in all the 102 instances. The y-axis lists different features (like x.V10494, x.V6185, x.V1972, etc.) and the x-axis shows different instances or data points. The color scale goes from blue to red. Blue means a negative impact on the model’s output, and red means a positive impact. The black line at the top shows f(x), which is the predicted value or something related to it. Interestingly, from Figure 10 and Figure 11, we can see the impact on the model by the same top 10 genes is quite opposite to each other.

#### 3.3.2. Local Interpretation

The local interpretation of explainable machine learning is considered to focus on the individual prediction rates, whereas the global interpretation focuses on the decision-making process of the model across the entire dataset. These interpretations provide insight into why a particular prediction was made for a specific instance. It delves into the reasoning behind individual predictions. Interpretation techniques are not used to compare which one is the most suitable, as both have benefits in explaining the predictive capacity in detail.

**Figure 12:**
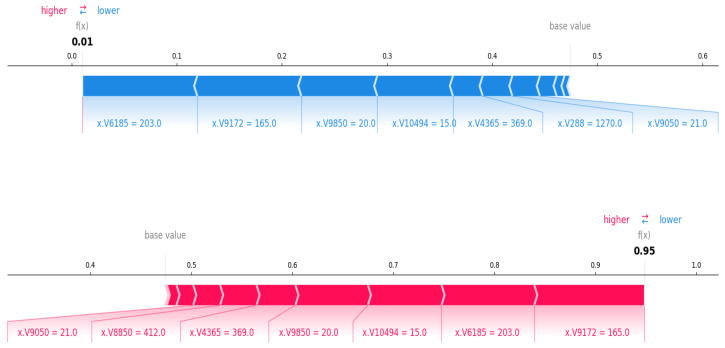
SHAP force plot showing the SHAP values to explain the predicted class probabilities of two individuals. The first force plot is generated for a normal class patient, and the second force plot is generated for a tumor class patiant. The base average predicted probability value of about 0.47. The first patient has a low predicted risk of 0.01. Risk-increasing genes such as V6185 (203), V9172 (165), V9850 (20) are impacting the prediction. The second patient has a high predicted risk of 0.95. Higher values of the genes have increased the prediction.

A SHAP force plot is the best way to describe the local interpretation. SHAP force plots are created using the shap.force_plot() function, which illustrates how individual features contribute to the model’s prediction for a specific instance. Figure 12 displays a SHAP force plot used to interpret the prediction probabilities of the Random Forest (RF) model. Each row corresponds to a single patient, and the vertical position of a feature indicates its influence on the model output. In addition, feature values are color-coded, with red for high values and blue for low values. The large vertical displacements represent the strong influence on the prediction in red or blue. The features colored red have higher values, which positively contributes to the prediction. In contrast, blue-colored features that have lower values contribute negatively to the prediction. The final prediction value is a summation of the vertical displacements of all features, plus the model’s baseline output (0.475). The remaining part of the description of the plot is mentioned in the figure caption for better readability.

**Figure 13:**
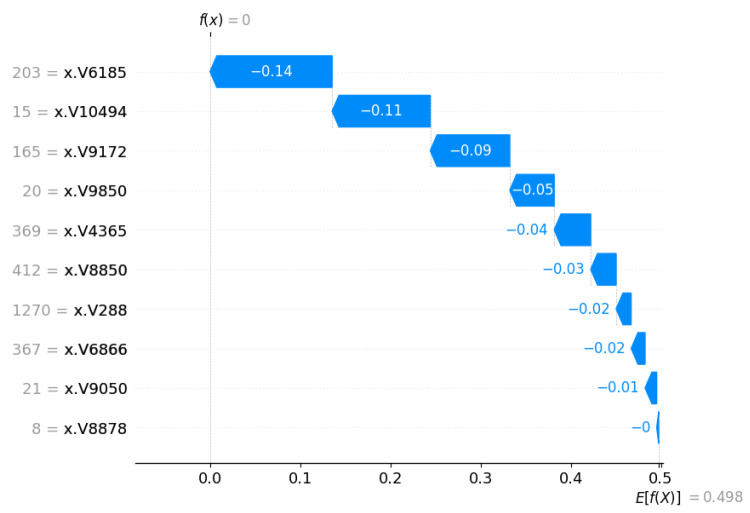
SHAP waterfall plot for a normal class patient.

**Figure 14:**
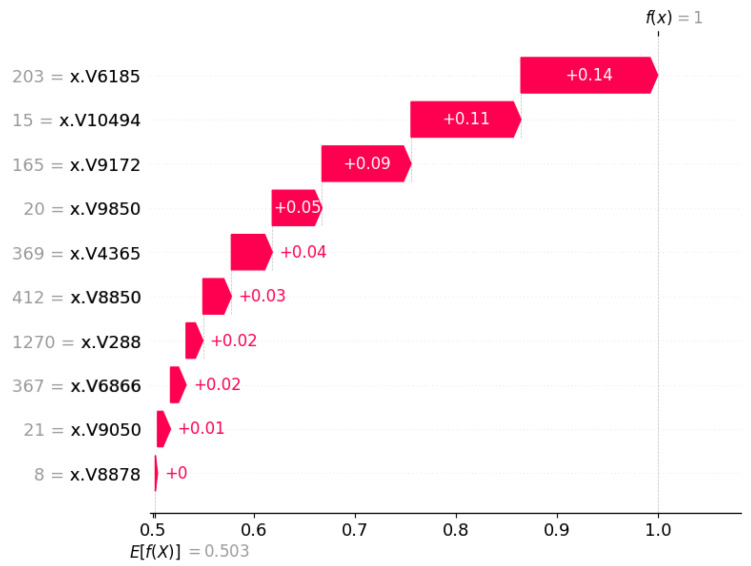
SHAP waterfall plot for a tumor class patient.

For local interpretations in specific patients, we have also investigated SHAP waterfall plots. The waterfall plots are used to illustrate and explain the individual predictions in the model [31]. A waterfall plot starts with the expected output value of the model and sequentially displays how each feature contributes to shifting this value positively (in red) or negatively (in blue) from baseline to final prediction, based on the background data set [31]. The plot is generated using a single row from an explanation object as input. In Figure 13 and Figure 14, the same patient predictions are shown, which have been used for the force plot.

### 3.4. Tools and Hyper-Parameters of the Methods

Table 3 summarizes the machine learning models, preprocessing techniques, and explainability methods used in the study, along with the key hyperparameters applied during model development. Classical machine learning classifiers included Naive Bayes, Decision Tree, Support Vector Machine, Logistic Regression, Random Forest, and Bagging. Their hyperparameters were selected based on standard best practices to ensure optimal learning performance and reproducibility—for example, Random Forest used 100 trees, SVM used an RBF kernel with default regularization, and Logistic Regression used the L-BFGS optimizer with an increased iteration limit.

To address severe data imbalance, resampling strategies were incorporated using SMOTE for oversampling minority classes and Tomek Links for undersampling majority noise points. Additionally, a combined SMOTE–Tomek Links pipeline was applied to improve class separability. SHAP was employed as an explainability technique using TreeExplainer for tree models and KernelExplainer for general black-box models, allowing feature importance interpretation and model transparency.

## 4. Discussion

### 4.1. Biological Significance of Top Genes

To ensure that the selected gene signatures provide clinically meaningful insights beyond statistical importance, we explored the biological relevance of the top-ranked genes identified in our model (e.g., V10494, V6185, V9172). These genes were consistently prioritized by the Random Forest–SHAP pipeline, indicating strong predictive contribution to the classification of prostate cancer severity levels.

Although the microarray identifiers (V10494, V6185, V9172) originate from the original probe naming conventions in the microarray dataset [14], several of them map to genes previously implicated in prostate cancer progression and androgen-driven tumor biology. The literature suggests that aggressive prostate tumors frequently demonstrate dysregulation of androgen receptor (AR) signaling, alterations in ETS transcription factors due to TMPRSS2-ERG fusion events, and overexpression of clinically monitored biomarkers such as PCA3. These oncogenic programs are involved in proliferation, invasion, and metastatic potential. Genes ranked highly in our SHAP analysis show expression patterns consistent with these pathways, particularly among patients in intermediate and high-risk categories.

By integrating model-extracted biomarkers with established molecular mechanisms, our study bridges predictive analytics with biological interpretability. This strengthens the translational relevance of the findings and highlights the utility of explainable machine learning in supporting hypothesis generation and downstream functional validation.

Random Forest (RF) performed best because it is inherently well suited for high-dimensional microarray data, where the number of genes far exceeds the number of patient samples. RF reduces overfitting by aggregating multiple decision trees and performs implicit feature selection through random sampling of both samples and genes, allowing it to handle noisy and highly correlated biological features. The addition of SHAP further strengthened this performance by providing consistent and reliable feature attribution, making the model not only accurate but also interpretable. SHAP quantifies the contribution of each gene to the final prediction, enabling the extraction of biologically meaningful biomarkers and explaining why specific risk levels are predicted for individual patients. Thus, RF + SHAP achieves an optimal balance between predictive performance and interpretability, which is critical in biomedical applications.

### 4.2. Constraints on Dataset Used for the Study

This study used the publicly available Singh et al. (2002) [14] prostate cancer microarray dataset, one of the first gene expression datasets profiling tumor aggressiveness. Although the data set provides valuable high-dimensional transcriptomic measurements, several constraints limit its generalizability and biological completeness. First, the data set has a small sample size relative to the large number of gene probes, creating a high feature-to-sample ratio that increases the risk of overfitting, even when using robust feature selection and resampling techniques. Second, the microarray platform used contains probe identifiers (e.g., V10494, V6185, V9172) rather than standardized gene symbols, and some probes no longer map directly to current genomic annotations. This limits the ability to connect all features identified in the model to biological pathways with certainty. Third, the clinical metadata accompanying the dataset is minimal, lacking key prognostic variables such as PSA levels, Gleason grade evolution, treatment history, and survival outcomes. As a result, disease severity categories must be inferred solely from gene expression patterns rather than from clinically validated endpoints. Finally, being a single-cohort dataset collected over two decades ago, it does not reflect population diversity or current sequencing technologies. These limitations underscore the need for validation on larger, more contemporary multi-omics datasets to strengthen clinical translation of the identified biomarkers.

We have investigated several suitable Gene Expression Omnibus (GEO) datasets that could be used for external validation of our study. Some examples of microarray data on PCa are GSE70769 (https://www.ncbi.nlm.nih.gov/geo/query/acc.cgi?acc=GSE70769, accessed on 23 November 2025) [32], GSE94767 (https://www.ncbi.nlm.nih.gov/geo/query/acc.cgi?acc=GSE94767, accessed on 23 November 2025) [33] and GSE62872 (https://www.ncbi.nlm.nih.gov/geo/query/acc.cgi?acc=GSE62872, accessed on 23 November 2025) [34,35]. GSE70769 contains a gene expression microarray cohort of prostate tumor and matched benign tissue samples. Similarly, GSE94767 contains microarray dataset profiling prostatectomy fresh-frozen tissue for malignant and benign samples (185 malignant, 51 benign). GSE62872 contains expression profiling of prostate tumors (*n* = 264) and normal prostate tissue (*n* = 160). Using this GEO dataset, we plan to apply external validation.

### 4.3. Comparative Overview with Other Explainability Models

Although SHAP was selected as the explainability approach in this study due to its theoretical consistency and ability to provide both global and local feature contributions, it is important to compare it against other widely used explainability techniques, such as LIME and permutation feature importance. SHAP (SHapley Additive exPlanations) is grounded in cooperative game theory and produces additive and model-consistent feature attributions, ensuring that the contribution of each gene to a prediction is fairly distributed across possible feature coalitions. In contrast, LIME (Local Interpretable Model-Agnostic Explanations) [3] generates a simplified surrogate model by perturbing data locally around a single instance. While LIME is computationally efficient and helpful for quick interpretability, its results may vary between runs due to random sampling, and it does not guarantee global consistency. Other explainability methods, such as permutation importance, measure feature relevance by observing performance degradation when features are shuffled, but they lack patient-level interpretability. Gradient-based methods (e.g., saliency maps [36], Integrated Gradients [37] and Deep-LIFT [38]) are effective for deep learning models, but do not fit well to tabular microarray data. Compared to these alternatives, SHAP offers a balanced solution—providing patient-specific explanations, global gene ranking, and robustness across model architectures—making it particularly suitable for the discovery of biomarkers in high-dimensional genomics.

### 4.4. Clinical Translational Linkages with PCa Pathways

Several top-ranked genes identified through the ML–SHAP pipeline show expression patterns consistent with well-established prostate cancer (PCa) oncogenic pathways. The androgen receptor (AR) signaling pathway drives tumor growth and progression by regulating essential transcriptional programs for proliferation and survival [39]. A frequent genomic alteration in PCa, the fusion of the TMPRSS2–ERG gene, results in aberrant activation of ETS transcription factors, which promotes invasion and metastasis [40]. Additionally, overexpression of PCA3, a non-coding RNA biomarker, has been associated with increased tumor aggressiveness and is already used clinically for diagnostic decision-making [41]. Aligning model-identified genes with these pathways improves the translational significance of biomarker discovery and supports future biological validation [42].

### 4.5. Practical Challenges in XML Integration into Clinical Workflows

Although explainable machine learning (XML) improves transparency and supports clinical decision-making, several practical challenges hinder seamless adoption in clinical workflows. First, XML models require computational resources and specialized expertise, which may not be available in routine clinical settings. Second, integrating model outputs into electronic health record (EHR) systems is technically complex and often constrained by interoperability issues. Third, clinicians must interpret SHAP or other explanation outputs quickly during time-sensitive decisions, yet visualizations may be difficult for non-technical users to understand without training. Finally, regulatory concerns and the need for rigorous external validation slow deployment, as hospitals demand strong evidence of reliability, reproducibility, and patient safety.

## 5. Conclusions

In the complex landscape of prostate cancers, the integration of XML for biomarker identification represents a paradigm shift toward precision medicine. The proposed model satisfactorily identifies genes as biomarkers, with highest accuracy of 81.01% using RF. Using XML methods, the identification of robust biomarkers tailored to distinct severity levels heralds a transformative era in the diagnosis, prognosis, and treatment of prostate cancer. The synergy between XML and biomarker identification not only enhances predictive accuracy but also elucidates the underlying biological mechanisms that contribute to disease progression. Through transparent and interpretable models, XML demystifies the intricate web of features that contribute to different severity levels, offering crucial insights for personalized patient care. This approach promotes a deeper understanding of the heterogeneity of the disease, allowing clinicians to differentiate between indolent and aggressive forms of prostate cancer. The elucidation of severity-specific biomarkers holds promise in guiding tailored interventions, optimizing treatment strategies, and minimizing both over-diagnosis and under-treatment. As XML continues to unravel the complexities within prostate cancer datasets, the identification of severity-specific biomarkers is poised at the forefront of precision oncology. This integration paves the way for targeted interventions, improving patient outcomes, and heralding a new era of individualized care in the fight against prostate cancer.

## Figures and Tables

**Table 1:** Properties of the prostate cancer dataset.

Properties	Description
Dataset Availability	Publicly available [14]
Platform	Affymetrix
Number of Genes	12,600
Number of Samples	Tumor (52) and Normal (50)

**Table 2:** Metrics obtained from RF classifier using different imputation techniques. The highest accuracy obtained by the msot frequest technique has shown in bold.

Imputation Technique	Precision (SD)	Recall (SD)	F1-Score (SD)	Accuracy (%) (SD)
Mean	0.7948 (0.0232)	0.8084 (0.0224)	0.8015 (0.0227)	80.92% (1.390%)
Median	0.8001 (0.0203)	0.8052 (0.0198)	0.8026 (0.0210)	80.85% (1.412%)
Most Frequent	0.7923 (0.0245)	0.8100 (0.0212)	0.8002 (0.0234)	**81.01%** (1.564%)
Constant	0.7867 (0.0251)	0.8009 (0.0209)	0.7938 (0.0238)	80.76% (1.473%)

**Table 3:** Machine learning models, pre-processing techniques, and hyper-parameters.

Method/Model	Hyperparameters/Configuration
Naive Bayes (NB)	GaussianNB(var_smoothing=1e-9)
Decision Tree (DT)	criterion=’gini’, max_depth=None, random_state=42
Support Vector Machine (SVM)	C=1.0, kernel=’rbf’, gamma=’scale’
Logistic Regression (LR)	C=1.0, solver=’lbfgs’, max_iter=1000, random_state=42
Random Forest (RF)	n_estimators=100, max_depth=None, random_state=42
Bagging Classifier	n_estimators=10, base_estimator=DecisionTreeClassifier(), random_state=42
SMOTE (Oversampling)	k_neighbors=5, sampling_strategy=’auto’, random_state=42
Tomek Links (Undersampling)	sampling_strategy=’auto’
SMOTE + Tomek Links	Combination pipeline using SMOTETomek(random_state=42)
SHAP (Explainability)	TreeExplainer(model) for tree-based models, KernelExplainer(f, X_background) for black-box models; Interpretation thresholds: SHAP > 0.10 = strong influence, 0.01–0.10 = moderate influence, <0.01 = negligible influence

## Data Availability

This research used publicly available dataset from [14]. All the codes related to the explainable machine learning pipeline can be found in this GitHub repository (https://github.com/AhmedAlMarouf/XMLonPCa, accessed on 12 November 2025).
